# Modeling Based on Ensemble Learning Methods for Detection of Diagnostic Biomarkers from LncRNA Data in Rats Treated with Cis-Platinum-Induced Hepatotoxicity

**DOI:** 10.3390/diagnostics13091583

**Published:** 2023-04-28

**Authors:** Zeynep Kucukakcali, Cemil Colak, Harika Gozde Gozukara Bag, Ipek Balikci Cicek, Onural Ozhan, Azibe Yildiz, Nefsun Danis, Ahmet Koc, Hakan Parlakpinar, Sami Akbulut

**Affiliations:** 1 Department of Biostatistics and Medical Informatics, Faculty of Medicine, Inonu University, 44280 Malatya, Turkey; zeynep.tunc@inonu.edu.tr (Z.K.); harika.gozukara@inonu.edu.tr (H.G.G.B.); ipek.balikci@inonu.edu.tr (I.B.C.); akbulutsami@gmail.com (S.A.); 2 Department of Pharmacology, Faculty of Medicine, Inonu University, 44280 Malatya, Turkey; onural.ozhan@inonu.edu.tr (O.O.);; 3 Department of Histology and Embryology, Faculty of Medicine, Inonu University, 44280 Malatya, Turkey; azibe.yildiz@inonu.edu.tr; 4 Department of Medical Biology and Genetics, Faculty of Medicine, Inonu University, 44280 Malatya, Turkey; danisnefsun@gmail.com (N.D.); ahmet.koc@inonu.edu.tr (A.K.); 5 Department of Surgery, Faculty of Medicine, Inonu University, 44280 Malatya, Turkey

**Keywords:** genomic, hepatotoxicity, machine learning, ensemble learning, biomarker

## Abstract

Background: The first aim of this study is to perform bioinformatic analysis of lncRNAs obtained from liver tissue samples from rats treated with cisplatin hepatotoxicity and without pathology. Another aim is to identify possible biomarkers for the diagnosis/early diagnosis of hepatotoxicity by modeling the data obtained from bioinformatics analysis with ensemble learning methods. Methods: In the study, 20 female Sprague-Dawley rats were divided into a control group and a hepatotoxicity group. Liver samples were taken from rats, and transcriptomic and histopathological analyses were performed. The dataset achieved from the transcriptomic analysis was modeled with ensemble learning methods (stacking, bagging, and boosting). Modeling results were evaluated with accuracy (Acc), balanced accuracy (B-Acc), sensitivity (Se), specificity (Sp), positive predictive value (Ppv), negative predictive value (Npv), and F1 score performance metrics. As a result of the modeling, lncRNAs that could be biomarkers were evaluated with variable importance values. Results: According to histopathological and immunohistochemical analyses, a significant increase was observed in the sinusoidal dilatation and Hsp60 immunoreactivity values in the hepatotoxicity group compared to the control group (*p* < 0.0001). According to the results of the bioinformatics analysis, 589 lncRNAs showed different expressions in the groups. The stacking model had the best classification performance among the applied ensemble learning models. The Acc, B-Acc, Se, Sp, Ppv, Npv, and F1-score values obtained from this model were 90%, 90%, 80%, 100%, 100%, 83.3%, and 88.9%, respectively. lncRNAs with id rna-XR_005492522.1, rna-XR_005492536.1, and rna-XR_005505831.1 with the highest three values according to the variable importance obtained as a result of stacking modeling can be used as predictive biomarker candidates for hepatotoxicity. Conclusions: Among the ensemble algorithms, the stacking technique yielded higher performance results as compared to the bagging and boosting methods on the transcriptomic data. More comprehensive studies can support the possible biomarkers determined due to the research and the decisive results for the diagnosis of drug-induced hepatotoxicity.

## 1. Introduction

The liver is involved in the realization of many chemical reactions, the production of bile, the metabolism of carbohydrates, lipids and proteins, and detoxification [1]. The liver is affected by drug-induced toxicities since it is responsible for the metabolism of several chemicals. We can list the agents that cause hepatotoxicity as natural toxic agents, chemical agents, drugs, and vitamins [2]. Hepatotoxicity can occur with many clinical conditions, such as acute and chronic damage, cirrhosis, and tumor observation. Drug-induced hepatotoxicity leads to cholestasis in hepatocytes and bile duct cells. Cholestasis causes intrahepatic accumulation of toxic bile acids and excretory products, which increases liver damage [3]. Drug-induced hepatotoxicity is a clinical condition that describes liver damage caused by therapeutic drugs, herbal products, and food supplements [4,5]. Drug-induced hepatotoxicity is one of the most important causes of patient mortality and morbidity and is a significant obstacle to new drug development studies. Therefore, it is among the serious health problems today [6]. Antineoplastic drugs, widely used in cancer treatment, cause unexpected effects while trying to destroy target cancer cells, and they can also damage and kill healthy cells [7]. Cisplatin (cis-diaminodichloroplatinum (II)), an antineoplastic drug, is one of the most known chemotherapeutic agents in cancer treatment. Hepatotoxicity, nephrotoxicity, neurotoxicity, testicular toxicity, and gastrointestinal disorders are the most common side effects limiting the use of cisplatin concerning the dose of service [8]. Using cisplatin in high doses can cause serious side effects in the liver, but it can also affect the individual’s hepatotoxicity. [9]. Lipid peroxidation, known as one of the essential mechanisms for liver damage caused by cisplatin, forms free oxygen radicals (ROS) released from oxidative stress. ROS plays a role in the emergence and pathogenesis of many diseases, such as heart diseases, toxic cell damage, and cancer, and makes an outstanding contribution to their emergence [10]. There are many conventional serum biomarkers used to assess hepatotoxicity. However, these biomarkers are not targeted at specific organs and can be affected by many conditions other than liver damage, such as muscle injury and pancreatitis [11,12,13]. There is a great need to find biomarkers to identify potential hepatotoxicity before clinical signs of drug-induced hepatotoxicity appear and provide an idea of whether a person will comply with treatment or exhibit liver failure. There is a great need to find biomarkers to identify potential hepatotoxicity before clinical signs of drug-induced hepatotoxicity appear and provide an idea of whether a person will comply with treatment or exhibit liver failure. In this sense, all omics technologies are promising in biomarker development. These techniques reveal the genetic profiles of the individual and enable the diagnosis of patients, treatment options, and drug doses to be determined individually [14]. In recent years, systematic genome studies have been conducted to understand the mechanism underlying cisplatin-induced hepatotoxicity. With innovations in RNA-seq technologies and computational biology, long non-coding RNAs (lncRNAs) have been rapidly identified and associated with the disease. Mutations in lncRNAs have been associated with many diseases, including cancer and neurodegenerative diseases. It has also been reported that any dysregulation of lncRNAs affects many normal cellular processes, such as proliferation, apoptosis resistance, and escape from tumor suppressors. Although the specific functions of lncRNA have not been clearly elucidated, it has been proven that liver diseases are associated with aberrant expression of lncRNAs [15,16,17]. However, genomic studies for drug-induced hepatotoxicity are limited, and studies related to lncRNA have not been conducted. Therefore, genomic studies are needed to overcome the deficiencies in this area.

Machine learning techniques, which have been widely used in the diagnosis of diseases and clinical decision support systems in recent years, are generally used in the disease prediction process in the health field. Machine learning, which has a very practical application in the health field, constitutes the basic infrastructure of applications, such as early diagnosis of cancer and chronic diseases, and determines the risk factors that cause them to occur [18,19]. The logic of ensemble learning, an important machine learning sub-field, is based on the idea that many classifiers can be combined to increase the correct prediction rate obtained by using a single base classifier. In other words, the ensemble learning method is based on the concept of merging several base classifiers to produce a more accurate and trustworthy model (meta classifier) than a base classifier (model) can achieve [20].

This study aimed to determine possible biomarkers for the diagnosis/early diagnosis of hepatotoxicity by modeling the transcriptomic data of liver tissues from rats treated with cisplatin hepatotoxicity and without pathology with ensemble learning methods.

## 2. Materials and Methods

### 2.1. Dataset

A total of 20 female Sprague-Dawley rats (age: 3 months; weight: 250 ± 20 g) were taken from İnönü University Experimental Animal Production and Research Center to detect possible biomarkers of drug-induced hepatotoxicity and to classify hepatotoxicity at the clinical level.

- Control group (C): The group in which cisplatin vehicle solvent was given intraperitoneally on the first day of the experiment.
- Hepatotoxicity group (CK): The group in which 7 mg/kg cisplatin was given intraperitoneally on the first day of the experiment.

On the 4th day of the experiment, liver tissue samples from rats treated with ketamine (225 mg/kg i.p.) and xylazine (24 mg/kg i.p.) were collected for transcriptomic and histopathological analyses under high-dose anesthesia.

### 2.2. Performing Histopathological and Immunohistochemical Analysis in Liver Tissue

#### 2.2.1. Histopathological Analysis

Tissues taken at the end of the experimental period were fixed in 10% formaldehyde. At the end of the fixation process, the tissues, which were washed with tap water, were subjected to dehydration and polishing processes, and embedded in paraffin. Sections of 4 µm thickness were taken from the paraffin blocks obtained afterward. The sections subjected to deparaffinization and rehydration procedures were stained using hematoxylin-eosin (H-E) for general evaluation. Stained preparations were examined with a Leica DFC-280 research microscope using the Leica Q Win Image Analysis System (Leica Micros Imaging Solutions Ltd., Cambridge, UK).

Histopathological evaluations were made by examining ten randomly selected areas in each section for hepatocyte degeneration (dense eosinophilic cytoplasm, pycnotic nucleus, hydropic degeneration) and sinusoidal dilatation. Each area examined was scored according to the severity of the changes as follows:

- 0 (no change);
- 1 (light);
- 2 (medium);
- 3 (severe) [21].

#### 2.2.2. Immunohistochemical Analysis

Sections subjected to deparaffinization and rehydration processes for immunohistochemical analyses were boiled in 0.01 M citrate (pH 6.0) using a pressure cooker for 15–20 min. To block the endogenous peroxidase enzyme activity on the sections to be examined, 3% hydrogen peroxide was applied for 12 min. Sections washed with Phosphate Buffered Saline (PBS) were subjected to protein blocking for 5 min. Afterward, the sections whose protein blocking process was completed were incubated with primary antibody (Hsp60) for 60 min at 37 °C. Biotin-based secondary antibodies were applied to the tissues washed with PBS for 10 min at 37 °C. At the end of this process, the sections were incubated with streptavadin peroxidase for 10 min at 37 °C. Then, after the chromogen application process was applied to the sections, they were stained with hematoxylin and closed with a water-based sealer. Immunohistochemical staining, the extent of immunoreactivity (0: no staining, 1: 1–2%, 2: 26–50%, 3: 51–75%, 4: 76–100%), and severity (0: no, +1: mild, +2: moderate, +3: severe) scored semi-quantitatively. The total staining score (H score) and the prevalence x severity was obtained by calculating [22].

Analyses were performed with a Leica DFC-280 research microscope using the Leica Q Win Image Analysis System (Leica Micros Imaging Solutions Ltd., Cambridge, UK).

### 2.3. Transcriptomic Analysis

#### 2.3.1. Isolation and Quality Control of Total RNAs from Tissue Samples

Total RNA isolation from tissue samples was performed using kits that provide high-efficiency isolation from low-volume samples. The miRNeasy Serum/Plasma Kit (Qiagen, Cat. No./ID: 217184) is designed to purify cell-free total RNA—primarily miRNA and other small RNA—from small volumes of serum and plasma. The used cDNA Synthesis Kit (RevertAid First Strand cDNA Synthesis Kit, Thermo Scientific, Waltham, MA, USA, Cat No. K1622) works with limited sample volumes, making it ideal for reversing valuable RNA into stable cDNA ready for accurate real-time quantification. The RNA amount was measured fluorometrically with Qubit (Qubit 3.0 Fluorometer, Life Technologies, Carlsbad, California, USA). In addition, the quality of RNAs was checked with Bioanalyzer before sequencing. Samples with RNA integrity number (RIN) ≥7 were sequenced.

#### 2.3.2. NGS Library Preparation and Sequencing for lncRNA Sequences

The lncRNA sequences were prepared using the “TruSeq Stranded Total RNA Library Prep Kit” from Illumina. Compatible with a wide range of samples, including low-quality DNA/RNA and formalin-fixed, paraffin-embedded (FFPE) samples, TruSeq Stranded Total RNA couples all the benefits of TruSeq RNA technology with Ribo-Zero ribosomal RNA reduction chemistry. The product enables analysis of coding and multiple forms of non-coding RNA with precise measurement of strand orientation, uniform coverage, and high-confidence discovery of features such as alternative transcripts, gene fusions, and allele-specific expression.

The sequences were prepared as follows:

- Ribosomal RNAs (rRNAs) were eliminated from total RNA, and the remaining RNAs were purified and fragmented.
- rRNA elimination was validated with the Bioanalyzer.
- Then, RNA fragments were reverse transcribed (first strand cDNA synthesis) using random hexamer sequences [23].
- Afterward, the RNA template was eliminated, and the second strand of cDNA (blunt ds cDNA) was synthesized [23].
- A single ‘A’ nucleotide was added to their 3’ ends to prevent the blunt ds cDNA fragments from binding together during the adapter ligation reaction.
- Indexing adapters were then added to hybridize the ds cDNA fragments to the flow cell surface.
- DNA fragments have been enriched.
- The libraries of the samples were normalized and pooled.
- Samples of 50M readings were made with the Illumina NovaSeq 6000 platform as a paired-end (PE) 2 × 150 base [24].

### 2.4. Data Analysis and Modeling Tasks

Whether the variables showed normal distribution or not was examined with the Shapiro–Wilk test. The data were presented as median (minimum-maximum) or mean±standard deviation (SD). The Mann–Whitney U test was used to compare non-normally distributed data, and the independent sample *t*-test was used to compare normally distributed data. The *p*-value < 0.05 was considered statistically significant. IBM SPSS Statistics 25.0 software was used in the analysis. The TMM (Trimmed mean of M values) normalization method was employed for the relevant data. In bioinformatic analysis, False Discovery Rate (FDR) was utilized for the assessments.

The elastic net variable selection method was used as the variable selection method within the scope of the study. R programming language-based RStudio was used in the implementation and variable selection stages of the bagging, boosting, and stacking ensemble learning models planned to be used in the study. This study used Acc, B-Acc, Se, Sp, Ppv, Npv, and F1-score metrics in the model performance evaluation. In addition, the graphics used in the visualizations were made using Excel software and R programming language.

## 3. Results

### 3.1. Histopathological Results

**Figure 1:**
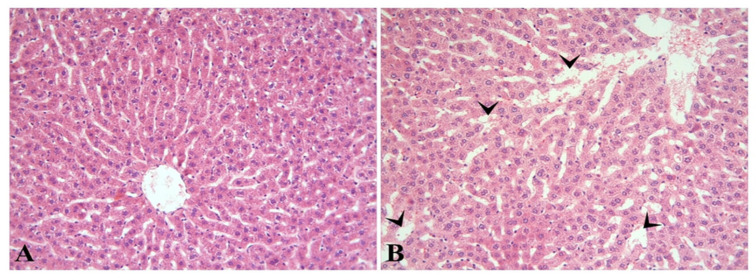
It is observed that the liver in the control group (**A**) has a normal histological appearance. Sinusoidal dilatation (arrowheads) is noticeable in the hepatotoxicity group (**B**). H-E; ×200.

The related liver damage was examined for hepatocyte degeneration and sinusoidal dilatation. In the sections where the H-E staining method was applied, the liver in the control group had a normal histological appearance, except for mild changes (Figure 1A). In the Hepatotoxicity group, hepatocyte degeneration was similar to the control group, while a significant increase in sinusoidal dilatation was observed (*p* < 0.0001) (Figure 1B). Histopathological evaluation results are given in Table 1.

### 3.2. Immunohistochemical Results

**Figure 2:**
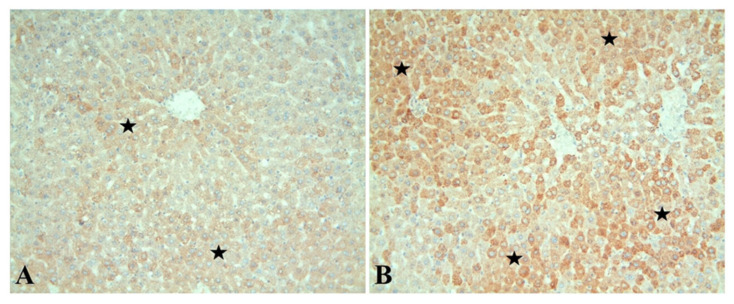
Hsp60 immunoreactivity in hepatocytes in the control (**A**) and hepatotoxicity (**B**) groups is shown with a star. Notably, the prevalence and severity of immunoreactivity were significantly higher in the hepatotoxicity group than in the control group. Hsp60 immunostaining; ×200.

Hsp60 immunoreactivity was distinguished by brownish staining in hepatocyte cytoplasm. Accordingly, it was observed that Hsp60 immunoreactivity was mild in the sections of the control group (Figure 2A). It was determined that cisplatin administration increased Hsp60 immunoreactivity in hepatocytes, and this increase was statistically significant when compared to the control group (Figure 2B) (*p* < 0.0001). The immunoreactivity evaluation results of the groups are given in Table 1.

Descriptive statistics for the rats used in the experiment are given in Table 2.

Descriptive statistics by the groups (control- hepatotoxicity) are given in Table 3.

### 3.3. Transcriptomic Analysis Results

The isolated RNA samples were examined with Agilent 2100 bioanalyzer system for quality control. The data whose quality control was completed were sequenced, and the quality control of the raw read data (fastqc) obtained as a result of sequencing was performed using FASTQC* software, version 0.11.8.

Quality-controlled raw read sequences were mapped to the reference genome using STAR* aligner software. The reference genome for mapping was taken from Rattus norvegicus (assembly mRatBN7.2) (GCF_015227675.2_mRatBN7.2_genomic.fna) and annotation track GCF_015227675.2_mRatBN7.2_genomic.gff_Lnc_rna. The quantification (count table) of reads mapped to the reference genome was conducted using the HTSeq* tool.

### 3.4. Differential Expression Results

The dataset used in the study contains 16,386 expressions. According to the results of the bioinformatics analysis, 589 (FDR < 0.05) lncRNAs showed different expressions in the groups. Of these, 450 showed up-expression (logFC > 1), and 139 showed down-expression (logFC < −1). Data set for bioinformatic analyses is presented in Supplementary Materials.

**Figure 3:**
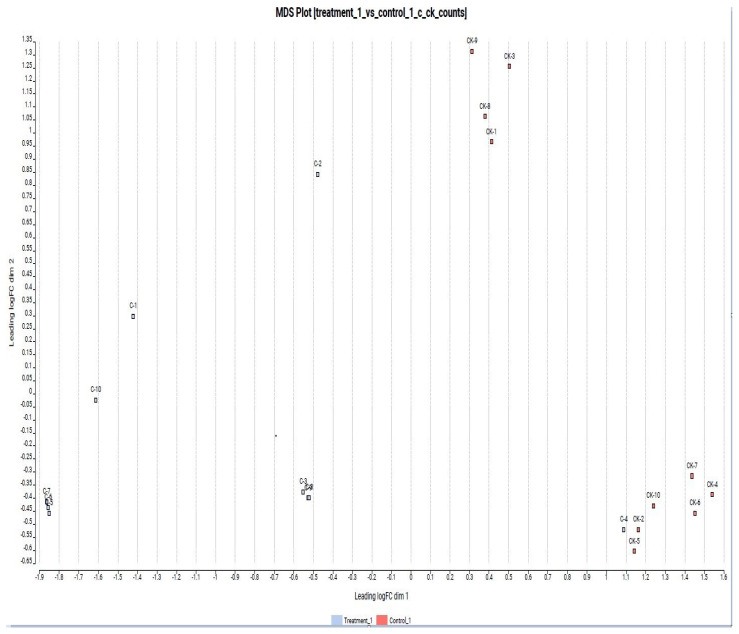
Hepatotoxicity group vs. control group comparison based on PCO analyses.

Based on the principal components (PCO) analysis, the distribution of the samples was found to be compatible in terms of lncRNA expression levels in the hepatotoxicity group (C) vs. control group (CK) comparison. Controls and application samples were collected in the same group. In this case, control and treatment samples presented similar expression level changes for similar lncRNAs. The visual figure for PCO analysis is given in Figure 3.

**Figure 4:**
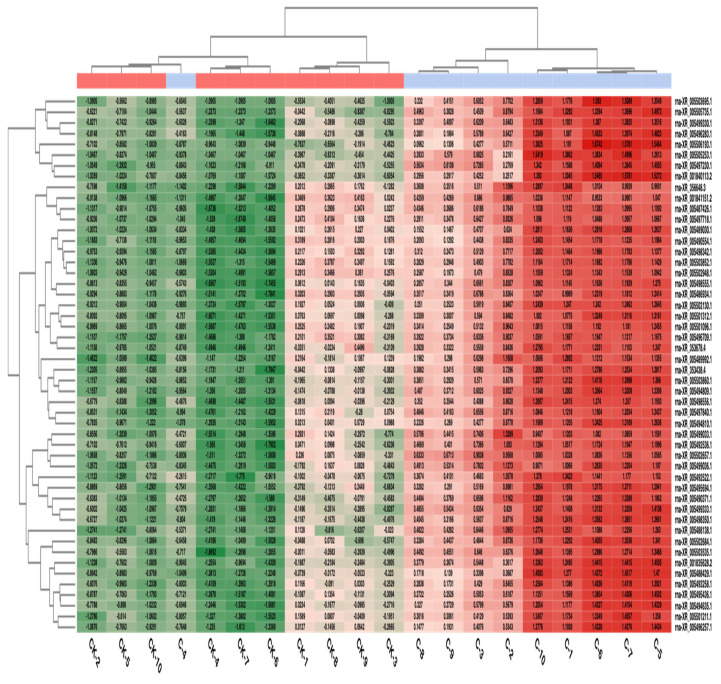
Heatmap for the fifty most expressed lncRNAs in two group comparisons.

The heatmap representation of the fifty most expressed lncRNAs in the two group comparisons is given in Figure 4.

In the C vs. CK comparison, it is observed that the application samples exhibited a different expression profile compared to the control, shown in red for the 50 lncRNAs that showed the most variation and in green for the suppressed expression level. The C-4 sample showed a different profile than the application samples.

**Figure 5:**
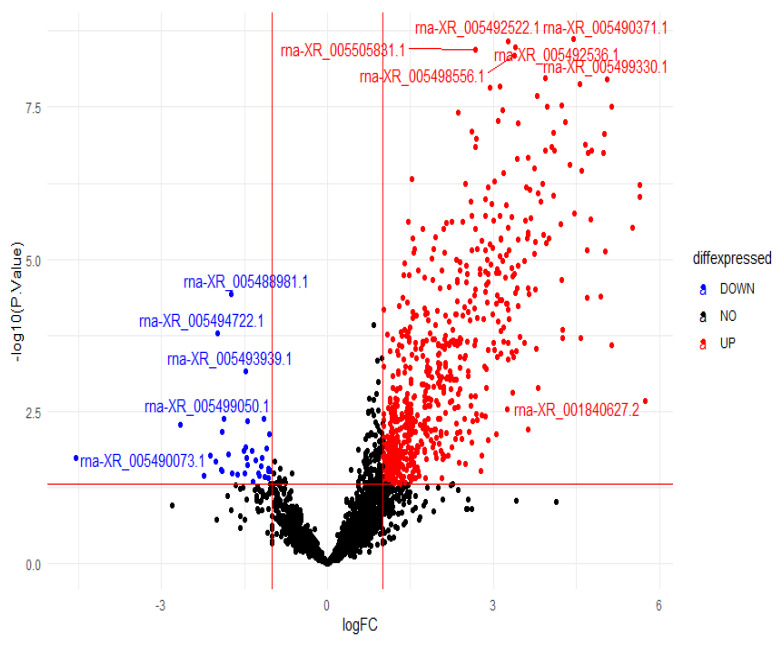
Volcano plot for differentially expressed genes in 2 group comparisons.

The volcano plot used to visualize differentially expressed genes is given in Figure 5. According to Figure 5, lncRNAs in red are up-regulated, and those in blue are down-regulated lncRNAs. The lncRNAs in black are the lncRNAs that are not expressed differently for the two groups.

### 3.5. Biostatistics Analysis and Modeling Results

The data of 16,386 lncRNAs in the data set were obtained by the TMM (Trimmed mean of M values) normalization method. From these data, 17 lncRNAs were chosen by the elastic net variable selection method from 589 lncRNAs with different regulations (up and down) between the two groups. The data analysis results of these chosen expressions are given in Table 4, which includes the selected expressions and descriptions of the data set, descriptors of the chosen expressions for the target variable under study, statistical significance, and the log fold change (LogFC) per gene for the target variable.

According to the statistical analysis results in Table 4, significant differences were detected between the groups in all lncRNA expressions (*p* < 0.05).

The results of performance metrics obtained as a result of bagging, boosting, and stacking models, which are ensemble learning models using selected lncRNAs, are given in Table 5.

According to the classification performance of the bagging, boosting, and stacking models, the bagging model’s Acc was 85%, B-Acc was 85%, Se was 70%, Sp was 100%, PPV was 100%, NPV was 76.92%, and F1-score was 82.4%. Acc of 85%, B-Acc of 85%, Se of 70%, Sp of 100%, PPV of 100%, NPV of 76.92%, and F1-score of 82.4% were obtained from the boosting model. Acc as 90%, B-Acc as 90%, Se as 80%, Sp as 100%, PPV as 100%, NPV as 83.3%, and F1-score as 88.9% were calculated from the stacking model.

**Figure 6:**
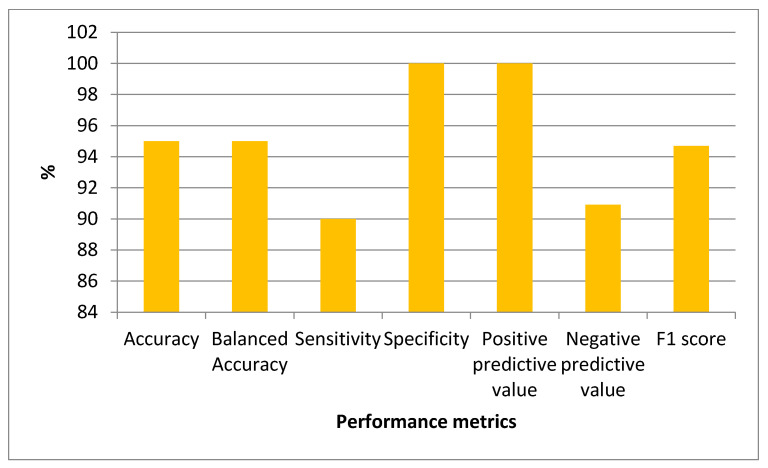
Graph of Performance Metrics for the Stacking Model.

The graph of the performance metrics for the stacking model, which gives the best result from the ensemble models used, is given in Figure 6.

**Figure 7:**
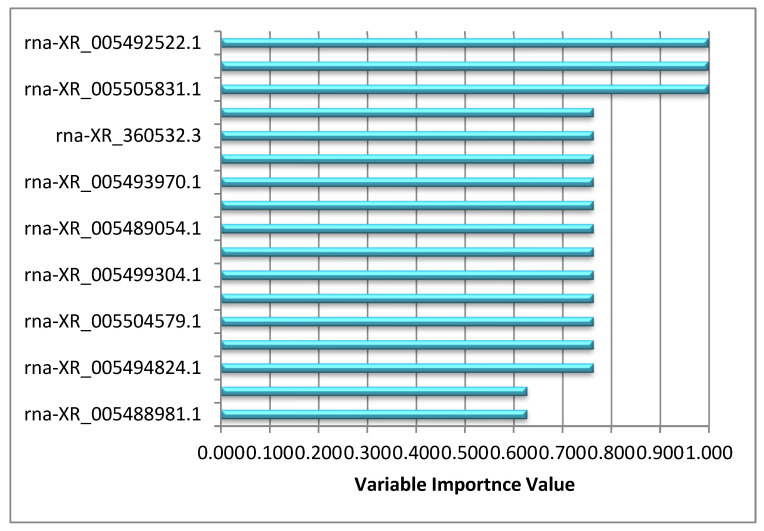
Graph of variable importance values obtained as a result of stacking model.

Figure 7 shows the variable importance levels of selected lncRNAs to explain the output variable.

## 4. Discussion

Liver toxicities based on drugs are known to be one of the leading causes of liver damage [2]. Drug-induced hepatotoxicities lead to various clinical manifestations, such as acute liver failure, cirrhosis, and liver cancer, which are non-specific changes [25]. The liver is an organ exposed to drug toxicities due to its functions and is personally affected [26,27]. Hepatotoxicity due to the amount of drug dose used is responsible for almost 50% of all acute liver failure cases, especially in the United States, England, and some Western countries [27]. It is essential to explain the mechanism of action of emerging liver toxicities and to develop treatment methods accordingly in terms of the mortality risk of patients [28]. Considering these factors, there is a need for non-known biomarkers that can explain drug-induced hepatotoxicity, which is a fundamental research direction and can reveal the damage to patients or predict whether the injury will develop. LncRNAs are molecules that interact with DNA, mRNA, protein, and miRNA structures to regulate and contribute to gene expression at epigenetic, transcriptional, post-transcriptional, and translational functional levels [29]. It has been reported that lncRNAs are involved in many regulatory mechanisms in the case of transcription and subsequent gene expression and perform primary functions for quite different biological processes [30]. Due to these mechanisms of action, studies with these RNAs have gained importance [31].

In the present study, transcriptomic and histopathological analyses were performed with liver samples taken from rats treated with cisplatin-induced hepatotoxicity and from rats in the control group.

As a result of histopathological analysis, liver damage was examined regarding hepatocyte degeneration and sinusoidal dilatation. In the sections where the H-E staining method was applied, the liver had a normal histological appearance except for mild changes in the control group. In contrast, hepatocyte degeneration was similar to the control group in the hepatotoxicity group, while a significant increase in sinusoidal dilatation was observed. In a previous study, a difference was observed in sinusoidal dilatation in the CIS group after HE staining was performed in the CIS platinum group [32]. Another study reported that the group in which CIS platinum was applied after HE staining showed massive hepatotoxicity compared to the control group. In addition, liver hepatocytes have been shown to show pycnotic nuclei with irregular nuclear membranes, while their cytoplasm contains vesicular rough endoplasmic reticulum and vestigial mitochondria with undifferentiated cisterns [33].

Heat shock proteins (HSPs) are a set of evolutionarily conserved molecules found in almost all living organisms [34,35]. HSP60 is a chaperone found in all mammalian cells and tissues, including the liver. This HSP performs many physiological functions not limited to its canonical cellular location in mitochondria [36,37]. It promotes mitochondrial protein folding and aids in the proteolytic degradation of denatured or abnormally folded proteins in an ATP-dependent manner [38]. HSP involves many physiological events but can be pathogenic in various conditions, including cancer and neurodegenerative diseases [39,40]. Variations in expression levels of HSP60 have been associated with multiple diseases and cancers, including hepatocellular carcinoma (HCC). Recent reports highlight the role and significance of HSP60 in human cancer development and management, whereby its targeting has produced promising therapeutic results [41,42]. It is known that cells rapidly produce several proteins known as heat shock proteins (HSP) and other different proteins in response to oxidative stress, which is observed as one of the essential mechanisms in liver damage caused by cisplatin [43,44]. According to immunohistochemical analysis, in the current study, Hsp60 immunoreactivity was distinguished by brownish staining in hepatocyte cytoplasm. Hsp60 immunoreactivity was mild in sections belonging to the control group. It was determined that cisplatin administration increased Hsp60 immunoreactivity in hepatocytes, and this increase was statistically significant compared to the control group.

This study used transcriptomic data obtained from liver tissues of rats treated with hepatotoxicity and control group rats for the relevant analysis. There are 16,386 lncRNA expressions in the acquired data set. According to the findings of the bioinformatics analysis, lncRNA with the id rna-XR_001840627.2 had a very high gene expression in the hepatotoxicity group compared to the control group. Similarly, rna-XR_005499199.1, rna-XR_005496955.1, rna-XR_005497501.1, rna-XR_001835923.2, rna-XR_005506070.1, rna-XR_005495875.1, rna-XR_005497501.1, rna-XR_005506070.1, rna-XR_005494195.1, and rna-XR_005503371.1, lncRNAs with id had very high gene expressions in the hepatotoxicity group compared to the control group. In an experimental study, lncRNAs were examined in MC-LR-induced hepatotoxicity, and the expression levels of three lncRNAs were found to be significantly increased in all treatment groups [45]. In another study, lncRNAs are known to play essential roles in chemical-induced adverse effects and liver disease as well as HCC. The change in the expression profile of hepatic lncRNAs was investigated in a mouse model exposed to 1,2-DCE [46].

lncRNAs with id rna-XR_005493896.1, rna-XR_005488982.1, rna-XR_005486345.1, rna-XR_005504727.1, rna-XR_005493990.1, rna-XR_005488390.1, rna-XR_005501078.1, rna-rna5490 -XR_005502677.1, and rna-XR_001837692.2 had low gene expression in the hepatotoxicity group compared to the control group.

According to the results of the biostatistics analysis performed with 17 lncRNAs selected by the elastic net variable selection method used in the study, a significant difference was found between the two groups for all lncRNAs, and the calculated OR values also support that these lncRNAs may be discriminative RNAs for the two groups. When the performance metrics obtained from the ensemble learning models using the selected 17 lncRNAs as input variables and taking the hepatotoxicity status as the target variable were examined, it was found that the stacking technique produced higher results than the other two methods. Performance metrics results show that the stacking method, one of the proposed models, can correctly classify hepatotoxicity when the high-performance metric values obtained in the classification of the two groups are considered. The rna-XR_005492522.1 (LOC120096269), rna-XR_005492536.1 (LOC120096276), and rna-XR_005505831.1 (LOC102557053) lncRNAs, which have the highest three significance values according to the significance of the variables obtained as a result of the stacking modeling, are for hepatotoxicity as a result of extensive studies and can be used as predictive biomarker candidates.

The present study may have some limitations. The results of the current study may illustrate changes in the lncRNA-based regulatory network associated with cisplatin treatment; however, it may have guiding potential for biomarker discovery and personalization of the treatment. From this aspect, it may be a limitation that this research is performed on experimental animals and forms a basis/background for further experimental research. In order to achieve the objectives examined in this study, experimental procedures could be performed on certain experimental animals due to restrictive factors. Therefore, more extensive experimental/clinical studies are necessary for the following periods.

## 5. Conclusions

Among the ensemble algorithms, the stacking technique yielded higher performance results as compared to the bagging and boosting methods on the transcriptomic data. For the potential biomarkers discovered from the current study to be used in the case of drug-induced hepatotoxicity, the achieved results need to be supported by different extensive studies. Once the accuracy of the obtained biomarker candidates is determined, possible treatment and diagnosis options can be personalized, and potential diagnostic procedures can be performed easily, quickly, and effectively after being confirmed in clinical trials.

## Figures and Tables

**Table 1:** Histopathological evaluation and Hsp60 immunoreactivity results.

Groups *	Hepatocyte Degeneration	Sinusoidal Dilation	Hsp60 Immunoreactivity
Control	0 (0–2)	0 (0–2)	2 (0–9)
Hepatotoxicity	0 (0–2)	1 (0–2) ^a^	4 (0–12) ^a^

^a^: a significant increase compared to the control group (*p* < 0.0001); * Med (Min-Max).

**Table 2:** Descriptive statistics for rats used in the experiment.

Variables	Mean ± SD
Rat weight starting (g)	230.2 ± 15.44
Rat weight end (g)	228.7 ± 14.974
Liver weight (g)	8.019 ± 0.844

**Table 3:** Descriptive statistics by groups (control-hepatotoxicity).

Variables	Control	Hepatotoxicity
Rat weight starting (g)	227.2 ± 19.96	233.2 ± 9.211
Rat weight end (g)	234.2 ± 16.705	223.2 ± 11.272
Liver weight (g)	7.92 ± 1.103	8.117 ± 0.515

**Table 4:** Descriptive Information on the Data Analysis Results.

Gene Name	Chromosome	ID	Group				LogFC	*p*
			CK		C			
			Mean ± SD	Median (Min-Max)	Mean ± SD	Median (Min-Max)		
LOC120094596	NC_051344.1	rna_XR_005488981.1	43.6 ± 15.665	39 (20–74)	12.9 ± 9.291	11.5 (0–32)	−2.58	<0.001 *
LOC120097437	NC_051351.1	rna_XR_005494824.1	0.6 ± 0.843	0 (0–2)	3.8 ± 1.033	4 (2–6)	2.04	<0.001 **
LOC120096352	NC_051348.1	rna_XR_005492760.1	1.8 ± 2.044	1 (0–5)	12.5 ± 5.126	13.5 (4–18)	2.38	<0.001 **
LOC120102815	NC_051340.1	rna_XR_005504579.1	1.5 ± 1.269	1.5 (0–3)	8 ± 3.712	8 (2–14)	1.80	<0.001 *
LOC120101756	NC_051338.1	rna_XR_005502657.1	31.8 ± 31.091	18.5 (7–108)	402.7 ± 151.646	424 (43–548)	3.34	<0.001 **
LOC120099881	NC_051336.1	rna_XR_005499304.1	4.7 ± 4.572	4 (0–14)	49.8 ± 18.66	55.5 (7–75)	2.93	<0.001 *
LOC102557053	NC_051341.1	rna_XR_005505831.1	54.3 ± 19.402	50 (28–89)	351.9 ± 115.959	364.5 (107–484)	2	<0.001 *
LOC103693406	NC_051345.1	rna_XR_005490393.1	8.9 ± 7.534	7 (1–23)	57.8 ± 17.536	63 (16–77)	2.29	<0.001 **
LOC120098296	NC_051353.1	rna_XR_005496472.1	3.9 ± 4.332	3 (0–14)	13.4 ± 5.358	13 (7–23)	1.38	0.001 **
LOC120094640	NC_051344.1	rna_XR_005489054.1	5 ± 5.85	2.5 (0–17)	28.6 ± 8.708	29 (9–38)	2.19	<0.001 **
LOC102552566	NC_051336.1	rna_XR_001836079.2	1.2 ± 0.919	1 (0–3)	9.6 ± 3.565	10 (3–16)	2.40	0.001 *
LOC120096990	NC_051350.1	rna_XR_005493970.1	2.1 ± 1.37	2.5 (0–4)	20.6 ± 8.669	21 (3–31)	2.69	<0.001 *
LOC120096276	NC_051348.1	rna_XR_005492536.1	213.2 ± 123.706	194.5 (64–482)	2101.1 ± 720.824	2139 (586–2927)	2.63	<0.001 *
LOC120099800	NC_051336.1	rna_XR_005499033.1	14.5 ± 15.58	8 (4–54)	168.9 ± 67.258	162.5 (49–310)	3.10	<0.001 **
LOC102553540	NC_051351.1	rna_XR_360532.3	1.4 ± 1.713	1 (0–5)	17.7 ± 7.273	19 (5–30)	2.99	<0.001 **
LOC120096269	NC_051348.1	rna_XR_005492522.1	5.4 ± 3.627	5 (0–11)	50.7 ± 11.47	55 (30–64)	2.67	<0.001 *
LOC120094133	NC_051343.1	rna_XR_005488138.1	2.7 ± 3.889	1 (0–12)	56.7 ± 21.386	60.5 (11–81)	3.98	<0.001 **

*: Independent sample *t*-test; **: Mann–Whitney U test; LogFC: Log fold change; C: hepatotoxicity group; CK: control group.

**Table 5:** Values for Metrics of Classification Performance of Bagging, Boosting, and Stacking Models.

Model	Metric	Value (%) (95% CI)
Bagging	Acc	85 (69.4–100)
	B-Acc	85 (69.4–100)
	Se	70 (34.75–93.33)
	Sp	100 (69.15–100)
	Ppv	100 (59–100)
	Npv	76.92 (56.39–89.57)
	F1-score	82.4 (65.6–99.1)
Boosting	Acc	85 (69.4–100)
	B-Acc	85 (69.4–100)
	Se	70 (34.75–93.33)
	Sp	100 (69.15–100)
	Ppv	100 (59–100)
	Npv	76.92 (56.39–89.57)
	F1-score	82.4 (65.6–99.1)
Stacking	Acc	90 (68.3–98.77)
	B-Acc	90 (68.3–98.77)
	Se	80 (44.39–97.48)
	Sp	100 (69.15–100)
	Ppv	100
	Npv	83.3 (59.14–94.53)
	F1-score	88.9 (75.1–100)

## Data Availability

The data set used in this study is given as a link in the Supplementary Materials of the article.

## Supplementary Materials

The following supporting information can be downloaded at: https://www.mdpi.com/article/10.3390/diagnostics13091583/s1, Table S1: Experimental study data file.
